# Evaluation of cervical lymph nodes using multispectral optoacoustic tomography: a proof-of-concept study

**DOI:** 10.1007/s00405-023-08073-y

**Published:** 2023-06-24

**Authors:** Christoph Becker, Johannes Hardarson, Andrea Hoelzer, Antje Geisler, Tobias Schulz, Charlène Reichl, Neil C. Burton, Tobias Schuler, Peter Kohl, Callum Zgierski-Johnston

**Affiliations:** 1 Department of Otorhinolaryngology-Head and Neck Surgery, University Medical Centre Freiburg, University of Freiburg, Killianstrasse 5, 79106 Freiburg, Germany; 2 iThera Medical GmbH, 81379 Munich, Germany; 3 Institute for Experimental Cardiovascular Medicine, University Heart Center Freiburg-Bad Krozingen, Freiburg, Germany; 4 Faculty of Medicine, University of Freiburg, Freiburg, Germany

**Keywords:** Multispectral optoacoustic tomography, Optoacoustic imaging, Optical imaging, Lymph node, Metastasis, Salivary gland, Thyroid gland, Head and neck cancer

## Abstract

**Objectives:**

Examination of lymph nodes is one of the most common indications for imaging in the head and neck region. The purpose of this study is to evaluate whether multispectral optoacoustic tomography can be used to observe chromophore differences between benign and malignant neck lymph nodes.

**Materials and methods:**

Proof-of-concept ex vivo study of resected cervical lymph nodes from 11 patients. The examination of lymph nodes included imaging with hybrid ultrasound and multispectral tomography system followed by spectral unmixing to separate signals from the endogenous chromophores water, lipid, hemoglobin and oxygenated hemoglobin; calculation of semi-quantitative parameters (total hemoglobin and relative oxygenation of hemoglobin). Comparison of the results from the hybrid measurement with the histopathological results.

**Results:**

Most patients suffered from squamous cell carcinoma (*n* = 7), also metastasis from salivary gland adenocarcinoma and papillary thyroid carcinoma, were included. The comparison between benign cervical lymph nodes and metastases showed significant differences for the absorbers water, lipid, hemoglobin and oxygenated hemoglobin and total hemoglobin.

**Conclusions:**

Our ex vivo study suggests that multispectral optoacoustic tomography can be used to detect differences between reactive lymph nodes and metastases. The measurement of endogenous chromophores can be used for this purpose. The examinations are non-invasively and thus potentially improve diagnostic prediction. However, potential influences from the ex vivo setting must be considered.

## Introduction

The first-line imaging for the examination of diseases in the head and neck region, such as lymph node enlargements or tumors of the thyroid and salivary glands, is ultrasound [1, 2]. With B-mode ultrasound examination, it is possible to obtain knowledge about the extent, margins and internal composition of the pathological structures, as well as about relationships to the surrounding area, such as infiltrative growth. Furthermore, examination of vascularization is possible with the help of doppler mode. Additional features such as elastography or contrast-enhanced ultrasound have been established and are used for certain indications, for example, thyroid nodules or parotid gland tumors [3, 4].

Ultrasound, like any imaging modality, cannot provide reliable evidence of malignancy. However, features of malignant tumors such as infiltrative growth into the surrounding area can already be detected with this examination technique. In case of suspected malignancy, the examination is often followed by surgical intervention, for example, needle biopsy or lymph node extirpation to exclude or detect malignant changes within these structures. Such surgical procedures inherently carry the risk of complications. Additional imaging that allows a more precise differentiation between benign or malignant changes would, therefore, be highly beneficial by allowing one to avoid surgical intervention.

**Fig. 1:**
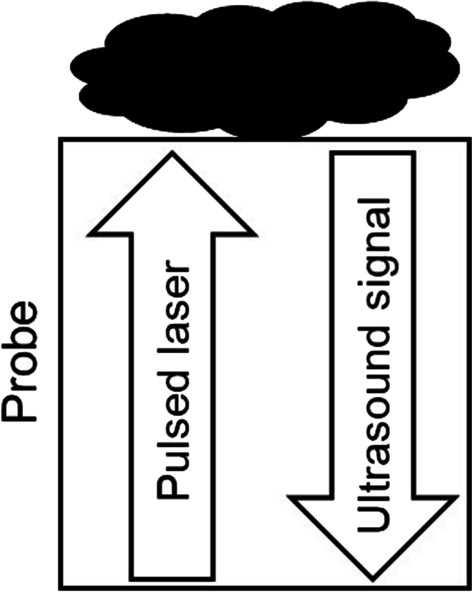
Schematic illustration of the MSOT probe. Tissue is illuminated with pulsed laser, transient thermoelastic expansion is detected by ultrasound

Optoacoustic imaging is based on the photoacoustic effect [5]. It is a comparatively new imaging technique that has been established, continuously developed and described in recent years [6–8]. The examined tissue is illuminated with pulsed laser light of different wavelengths in the near-infrared range. The light is absorbed by specific components in the tissue, such as water, hemoglobin or fat. Transient heating and the resulting thermoelastic expansion generate pressure waves that are detected by an ultrasound sensor (Fig. 1). Thus, MSOT can generate images of the optical absorption across the tissue at each selected wavelength.

These selected wavelengths are then used by an online algorithm allowing live preview of specific endogenous and exogenous chromophores through spectral unmixing.

Moreover, hybrid multispectral optoacoustic tomography (MSOT)/ultrasound systems are able to use this spectral unmixing to map the distribution of these chromophores to the underlying structure as visualized by ultrasound. Such an approach has been used for lymph node examinations in melanoma, detection of collagen for the monitoring in muscular dystrophies, or measurement of inflammatory activity in inflammatory bowel disease [9–11].

The evaluation of a cervical lymphadenopathy is challenging. There are various reasons for enlarged neck lymph nodes with the spectrum ranging from reactive nodes in the context of infectious diseases, autoimmune diseases, or lymphoma to metastasis of head, neck, or thyroid carcinoma. An imaging technique that can reliably distinguish between reactive and malignant lymph nodes is necessary.

The aim of this study is to evaluate whether cervical lymph nodes can in principle be visualized using a hybrid MSOT/ultrasound system and whether a differentiation between reactive and malignant lymph nodes is possible.

## Methods

The study was performed in accordance with the guidelines of the Helsinki Declaration of 1975, as revised in 2013 and the study protocol was approved prior to data collection by the local institutional review board and registered on German Clinical Trials Register. All patients provided written informed consent before study inclusion.

## Patients

All patients underwent at least routine ultrasound diagnostics (Canon Model TUS-AI800, Canon Medical Systems Corporation, Tochigi, Japan), in some cases supplemented by further imaging procedures such as computed tomography. Based on these imaging results, the indication for surgical resection was made. Patients with reactive lymph nodes and patients with metastasis of different carcinoma were enrolled in the study. The complete samples were examined with MSOT and subsequently analyzed histopathologically. The results of the MSOT were then subsequently matched to the corresponding results of the histopathologic examination.

## Study design

Since the available hybrid ultrasound/MSOT device (MSOT Acuity Echo research system, iThera Medical, Munich, Germany) was intended for research purposes without approval by a notified body for use on patients, the study was conducted as a proof-of-concept ex vivo investigation. Several studies have already demonstrated the ex vivo feasibility of optoacoustic tomography [8, 12, 13]. The integrated ultrasound is used for better anatomical orientation during subsequent image evaluation. In all cases, resection was performed first, followed immediately by MSOT examination of the specimen within 20–50 min. To create a situation comparable to clinical practice, no further manipulation of the specimen, such as cleaning, was performed after resection. After MSOT measurement, the specimen were fixed in formalin and sent to the Institute of Pathology.

At the outset of the study, there was discussion within the study group as to whether the ex vivo setting and measurements within 40 min of resection might have an impact on the results. Because other studies with larger numbers of cases already described ex vivo measurements without evidence of changes at least in the short-term period after resection, we did not see any absolute limitations in this regard with respect to our study setting [9, 13, 14]. It appears that relevant changes in tissue morphology occur later. Nevertheless, a preliminary test series was carried out first to establish our own experimental setup and thus confirm the observations of other ex vivo studies. We, therefore, evaluated whether when measuring ex vivo the values for the above parameters underwent relevant changes over a period of up to 40 min. Tissue scans from two parotid gland tumors and one lymph node were performed every 20 min in three different specimens (i.e., measurements at 0, 20 and 40 min). For the preliminary test series, two parotid gland tumors and one lymph node were examined. There were no significant differences for all absorbers mentioned above (*p* > 0.05; Table 1).

**Table 1:** Results of the preliminary test series; MPI (mean pixel intensity) for the different absorbers

Chromophore	MPI (mean and standard deviation)		
	0 min	20 min	40 min
H_2_0	51.64 (18.5)	38.85 (44.8)	44.10 (50.8)
Hb	0.006 (0.001)	0.005 (0.005)	0.004 (0.004)
HbO_2_	0.052 (0.023)	0.035 (0.018)	0.027 (0.016)
Lipid	507.32 (281.9)	275.58 (389.5)	328.53 (449.8)
HbT	0.058 (0.024)	0.039 (0.023)	0.032 (0.020)
mSO_2_	0.827 (0.018)	0.794 (0.065)	0.802 (0.041)

*H*_*2*_*O* water, *Hb* hemoglobin, *HbO*_*2*_ oxygenated hemoglobin, *HbT* total hemoglobin, *msO*_*2*_ relative oxygenation of hemoglobin

**Fig. 2:**
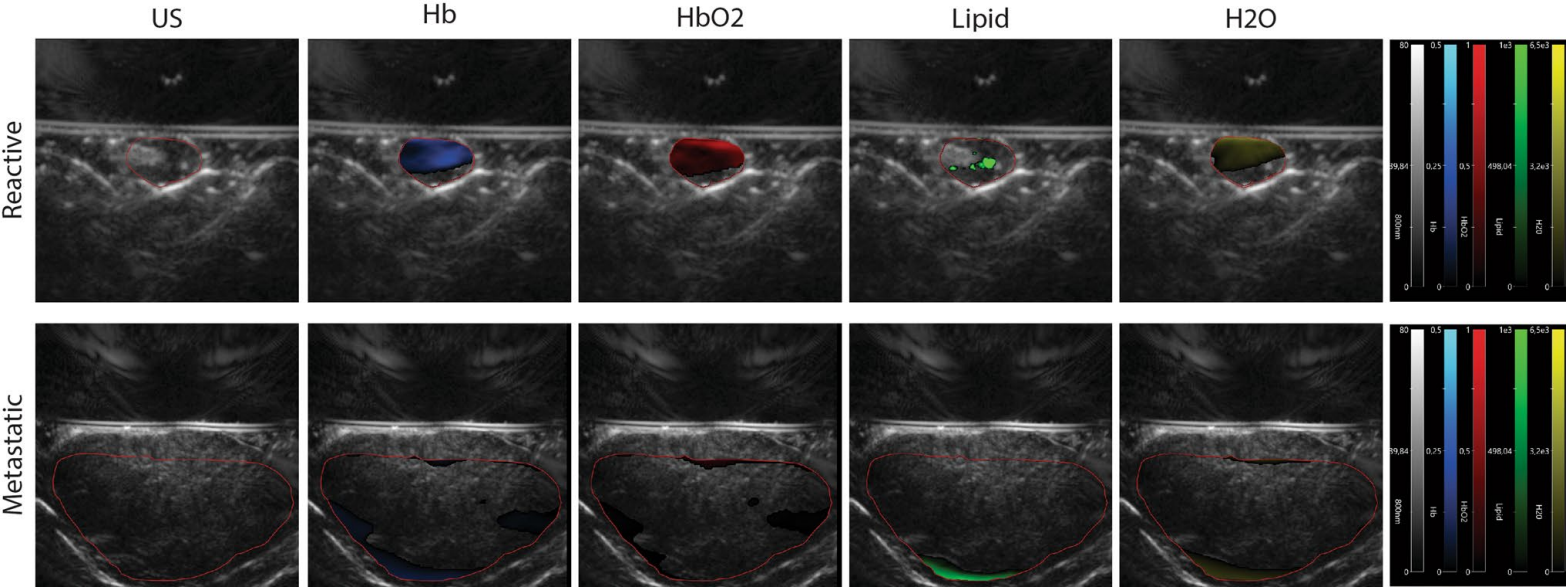
Visualization of the unmixed chromophores (Hb, HbO_2_, lipid, water) in a reactive and a metastatic lymph node. Region of interest shown in ultrasound image (left column)

**Fig. 3:**
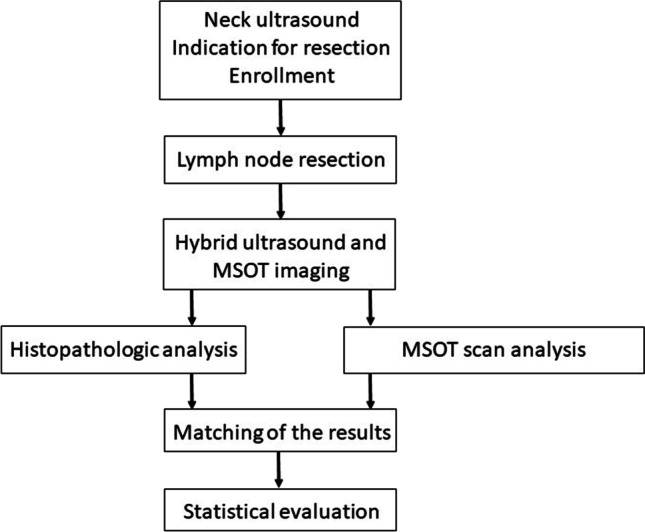
Illustration of the study workflow

For MSOT imaging, the specimen was positioned with ultrasound gel on the hybrid ultrasound/MSOT probe. MSOT images were acquired at 15 different wavelengths (680, 700, 715, 730, 750, 760, 780, 800, 830, 850, 870, 900, 920, 950 and 1210 nm). Wavelengths were chosen to allow measurement of previously selected endogenous chromophores. The duration of the complete examination took about 10 min. For image reconstruction and analysis, the software ViewMSOT (iThera Medical, Munich, Germany) was used. For the final figure, images were processed using ImageJ to highlight each unmixed chromophore inside the region of interest (Fig. 2). Regions of interest in the scan were defined using ultrasound. Spectral unmixing was used to separate signals from water (H_2_O), lipid, hemoglobin (Hb) and oxygenated hemoglobin (HbO_2_). Semi-quantitative parameters were extracted following unmixing of data; these parameters were total hemoglobin (HbT = Hb + HbO_2_) and relative oxygenation of hemoglobin (msO_2_ = HbO2/HbT). An overview of the study workflow is shown in Fig. 3.

### Statistical analysis

Statistical analyses were performed using IBM Statistical Package Social Sciences (SPSS) version 23 software (Chicago, Illinois, USA). For group comparisons, the t test for equally distributed and the Mann–Whitney test for non-equally distributed groups were used. Probabilities of less than 0.05 were accepted as statistically significant.

## Results

11 patients (8 male/3 female, median age 67 years) were included in the study. Ten patients underwent a neck dissection, one patient a partial parotidectomy for a suspected tumor of the parotid gland. Subsequent to resection, lymph nodes suspected of being metastatic were separately identified for further hybrid ultrasound and MSOT measurements. Furthermore, the remaining neck level specimen were also examined using MSOT. All lymph nodes that were preoperatively detected as enlarged by ultrasound could also be visualized by the hybrid ultrasound/MSOT system.

The examined lymph nodes were classified as metastasis (*n* = 21) or benign (*n* = 4) lymph node depending on the histopathological result. It was, therefore, possible that metastases and benign lymph nodes were examined from the same patient. Each lymph node was measured at different positions to obtain representative values for the entire specimen in each case.

Most patients suffered from metastases of a squamous cell carcinoma of the upper aerodigestive tract (*n* = 7). Furthermore, two patients with metastases of a papillary carcinoma of the thyroid gland, one patient with metastases of an adenocarcinoma of the parotid gland and one patient with a reactive lymph node of the parotid gland were included. Patient and tumor characteristics are summarized in Table 2.

**Table 2:** Patients and tumor characteristics

Patient number	Sex	Age	Primary tumor localization	Tumor entity	Stage (pTN)	Number of metastases	Extracapsular spread	Composition (ultrasound)
1	Male	80	Oropharynx	SCC, HPV +	T2N1	1	No	Solid
2	Male	77	Thyroid gland	Papillary carcinoma	T1bN1b	2	Yes	Mixed cystic/solid
3	Male	64	Larynx	SCC	T2N0	0	N/A	N/A
4	Male	47	Oropharynx	SCC, HPV +	T2N1	2	No	Solid
5	Male	67	Oropharynx	SCC, HPV +	T2N1	2	No	Solid
6	Male	87	Thyroid gland	Papillary carcinoma	T1aN1b	6	Yes	Solid
7	Male	56	Oropharynx	SCC, HPV +	T2N1	3	No	Solid
8	Male	83	Oral cavity (initially)	SCC	T0N3b	2	Yes	Mixed cystic/solid
9	Female	49	Oropharynx	SCC, HPV +	T2N1	4	Yes	Mixed cystic/solid
10	Female	81	Parotid gland	Adenocarcinoma	T4aN3b	2	Yes	solid
11	Female	48	Parotid gland	Benign/reactive LN	N/A	0	N/A	N/A

*SCC* squamous cell carcinoma, *HPV* human papillomavirus, *N/A* not applicable

To increase the significance of the study, we grouped all lymph nodes in “reactive lymph nodes” and “metastatic lymph nodes”. The mean pixel intensities for the different absorbers and *p* values are shown in Table 3. There are significant differences between metastatic and reactive lymph nodes for water, lipid, hemoglobin and oxygenated hemoglobin and total hemoglobin. Lymph node metastases showed higher levels than reactive lymph nodes for all these chromophores. The only absorber without significant difference was the semi-quantitative parameter “relative oxygenation of hemoglobin”.

**Table 3:** Mean pixel intensity for the different absorbers

Absorber	Mean pixel intensity	Standard deviation	*p* value
H_2_O			
Metastatic LN	104.87	66.55	0.002*
Reactive LN	382.75	87.54	
Hb			
Metastatic LN	0.02	0.01	0.003*
Reactive LN	0.06	0.03	
HbO_2_			
Metastatic LN	0.03	0.02	0.004*
Reactive LN	0.09	0.05	
Lipid			
Metastatic LN	174.70	239.75	0.038*
Reactive LN	621.54	421.22	
HbT			
Metastatic LN	0.04	0.02	0.002*
Reactive LN	0.15	0.05	
mSO_2_			
Metastatic LN	0.59	0.12	0.553
Reactive LN	0.61	0.14	

*Probabilities of less than 0.05 were accepted as statistically significant

*H*_*2*_*O* water, *Hb* hemoglobin, *HbO*_*2*_ oxygenated hemoglobin, *HbT* total hemoglobin, *msO*_*2*_ relative oxygenation of hemoglobin, *LN* lymph nodes

## Discussion

MSOT is a relatively new and emerging imaging method. It allows noninvasive imaging for different pathologies. With a penetration depth of up to 2.5 cm, it is well-suited for use in the head and neck area [9].

Histopathologic classification of carcinomas or their metastases is based primarily on morphologic criteria, for example, specific cell types, grade of differentiation, or growth patterns, such as perineural invasion [15]. The further characterization is usually done by immunocytochemistry and molecular pathology [15]. MSOT, on the other hand, allows tumor characterization based on “static” tissue components such as lipid or water on one hand, and on physiological differences such as the proportion of oxygenated hemoglobin on the other hand.

Alterations in lipid metabolism play an important role in tumor development and metastasis [16]. These changes were shown to increase the energy available to tumor cells, improve survival through anti-oxidative and anti-apoptotic mechanisms, and modulate the immune response to tumor cells [16]. Although the metastatic lymph nodes in our study showed a lower lipid content than the reactive lymph nodes, this does not contradict the cited findings, as the changes are due to lipid metabolism and do not necessarily require a higher lipid content. Rather, the lower lipid content was expected, as necrotic areas, for example, may be present within metastases. The reduced lipid content in malignant tissue can also be observed in the comparison of benign and malignant thyroid nodules [17].

Due to the usual histopathological examinations of lymph nodes described above, the higher water content of reactive lymph nodes in the MSOT measurement can only be integrated into the context of existing characterizations to a limited extent. Here, too, changes due to areas of necrosis are a possible explanation. The determination of water content has so far been described in a few studies in which magnetic resonance imaging (MRI) was used as the imaging method. Wang et al. evaluated retropharyngeal lymph nodes in patients with (suspected) nasopharyngeal carcinoma using different MRI-derived relaxometry parameters [18]. Here, a higher water content in metastases or metastasis-specific lymph nodes was observed. However, the results seem to be comparable only to a limited extent, on one hand due to the different modalities, and on the other hand due to the fact that the retropharyngeal lymph nodes were classified as metastases only on the basis of size, shape and proven nasopharyngeal carcinoma and not—as in our study—on the basis of a histopathological examination.

A known characteristic that influences the therapy and thus also the prognosis of a carcinoma is the presence of tumor hypoxia [19]. Various mechanisms are responsible for the extent of hypoxia, including the degree of vascularization, general anemia or factors, such as smoking, which can influence the oxygen concentration in the tissue [20, 21]. In our study, metastases showed both lower levels for hemoglobin and oxygenated hemoglobin. Various imaging techniques are used to assess the degree of necrosis or hypoxia in tumor tissue, for example [18F]-fluoromisonidazole-PET [22, 23]. However, these techniques are associated with higher equipment costs or exposure to radiation; where appropriate, MSOT could represent a more feasible alternative.

The lower MSOT values for hemoglobin could also be related to neoangiogenesis. Naresh et al. compared neoangiogenesis in head and neck carcinomas, reactive and metastatic lymph nodes in a study and were able to show that there is abundant native vascularization in reactive lymph nodes and comparatively less vascular proliferation in metastases [24].

Initial head and neck examinations using MSOT were performed for pathologies of the thyroid gland. In a pilot study, Roll et al. showed differences between patients with Graves’ disease and healthy patients and between benign and malignant tumors, respectively [17]. Thus, MSOT can thereafter potentially provide additional information with respect to both thyroid nodule characterization and thyroid function. With focus on thyroid cancer, Kim et al. proposed a combined risk stratification for thyroid nodules using the American Thyroid Association ultrasound classification system and photoacoustic imaging [25]. This combination led to a new scoring method with a sensitivity of 83% and a specificity of 93% and could, therefore, according to the authors, improve the diagnosis of thyroid malignancies and avoid unnecessary fine-needle biopsies.

The value of MSOT in the diagnosis of metastases of malignant melanoma has been known for some time. Due to the visualization of the chromophore melanin, it is possible to identify metastases [9]. In a cross-sectional study, Stoffels et al. identified sentinel lymph nodes using the exogenous chromophore indocyanine green, which was injected peritumorally [26]. Compared to the gold standard (technetium marked sentinel lymph nodes) the intraoperative concordance rate was 96.4% [26]. The detection of metastases in amelanotic melanomas and the differentiation to melanin in normal cells is challenging [27]. Therefore, MSOT offers a potential benefit in diagnostics and therapy in malignant melanoma [28].

There are no comparable specific chromophores such as melanin in other carcinomas. In addition to indocyanine green, several other exogenous chromophores have been used for the diagnosis of lymph nodes. Song et al. used methylene blue in an in vivo rat model for identification of sentinel lymph nodes [29]. The advantage of indocyanine green and methylene blue is that these substances are approved for use in patients.

More experimental substances such as gold-based particles may provide advantages by improved targeting of specific tumor features [30]. A recent study by Vonk et al. investigated the use of MSOT in the diagnosis of metastases from head and neck cancer [31]. The epidermal Growth Factor Receptor (EGFR)-targeted fluorescent tracer Cetuximab-800CW was used in the study. As a result, different distributions of EGFR were observed between benign lymph nodes and metastases. Similar results were obtained by Nishio et al. who assessed photoacoustic molecular imaging of the antiepidermal growth factor receptor antibody (panitumumab) conjugated to a near-infrared fluorescent dye (IRDye800CW) [32]. After examining 53 lymph nodes, the authors concluded that photoacoustic imaging can be used to detect occult lymph node metastases.

However, the application of an exogenous chromophore—especially when non-standard preparations are used—fundamentally limits the feasibility of MSOT. In their study, Luke et al. investigated whether distinctions between benign and metastatic lymph nodes can be made on the basis of other endogenous chromophores [33]. In a mouse model of oral cancer, they found differences in the oxygen saturation (sO_2_) with lower saturation in seven metastatic lymph nodes.

Differentiation of metastases and benign lymph nodes using endogenous chromophore offers several advantages. On one hand, measurement is possible with the devices already approved for use on patients. However, we had to forgo this option for our study, as only a device modified for scientific purposes was available. On the other hand, with a more precise pre-therapeutic/preoperative imaging, both better patient counseling and therapy planning are possible. For example, if intraoperative findings are used to decide the extent of surgical resection, MSOT may be a potential alternative to fresh frozen section. Follow-up studies comparing intraoperative lymph node samples with frozen sections are, therefore, useful. The value of MSOT in the diagnosis of lymphoproliferative diseases should also be evaluated.

Our study has several limitations. The general availability of MSOT is limited, not least for cost reasons. In our particular case, the device was not CE certified and, therefore, not approved for use on patients. For the study design of a proof of concept study, this was feasible; however, the results cannot necessarily be transferred to in vivo conditions. Initial tissue changes from the time of resection to the recording are possible, especially regarding blood oxygenation. The fact that numerous other studies also perform measurements in the ex vivo setting, in some cases even without specifying the measurement timepoints, does not necessarily mean that there are already early changes in the tissue. In particular, interpretations concerning chromophores that are subject to natural variations in the in vivo situation, such as the oxygenation of hemoglobin, should be made with caution in ex vivo studies. Especially when comparing the parameters Hb/HbO_2_ with other ex vivo studies, fluctuations in the measured values (mean pixel intensity) are noticeable. For example, while in our study a higher mean pixel intensity for Hb/HbO_2_ was observed in reactive lymph nodes, ex vivo studies in other tumour entities show opposite results [34]. Although the study settings are formally comparable, other (external) influencing factors could limit the comparability of different studies [14, 17].

In addition to the low number of cases, the standard deviations could be caused by the number of cases and technical limitations. Furthermore, follow-up studies must first confirm that the differentiation of healthy and tumor tissue on the basis of the chromophores mentioned and available is possible. It is possible that the advantages of the technique lie less in the determination in the entity and more in the potential to enable biological characterization, for example, in the assessment of hypoxic/non-hypoxic metastasis.

The majority of included metastases originated from human papillomavirus-positive squamous cell carcinomas. Frequently, these carcinomas present with (predominantly) cystic metastases. However, in our collective, there was no completely cystic metastasis. To increase the power of this rather experimental study, a simplified distinction was made between benign lymph nodes and metastases (regardless of origin). For a proof-of-concept study, this is sufficient for now—but follow-up studies focusing on specific entities should be sought.

## Conclusion

Our data suggest that MSOT is able to detect differences in the intensity of endogenous chromophores between reactive and metastatic cervical lymph nodes. The advantages of this method are that it can be used non-invasively and can provide further information about the examined tissue, such as the content of water, lipid or hemoglobin. The improved imaging could also have an impact on the pre-therapeutic characterization of cervical lymph nodes as well as the assessment under treatment, for example, in the context of radiation therapy. However, the results of this rather experimental study need to be further evaluated in studies with larger numbers of cases and in vivo settings.

## Data Availability

Not applicable.
